# Preparation and Properties of Reprocessable Hydrogenated Styrene–Butadiene Rubber

**DOI:** 10.3390/polym18060688

**Published:** 2026-03-12

**Authors:** Tianxi Li, Chaolun Pan, Dongmei Yue

**Affiliations:** Key Laboratory of Beijing City on Preparation and Processing of Novel Polymer Materials, Beijing University of Chemical Technology, Beijing 100029, China

**Keywords:** hydrogenated epoxidized styrene–butadiene rubber (HESBR), reprocessability, dynamic bond

## Abstract

Styrene–butadiene rubber (SBR) is one of the most widely used synthetic elastomers. However, the unsaturated C=C bonds in its backbone limit its long-term stability under harsh service conditions. Furthermore, conventional sulfur vulcanization forms irreversible covalent crosslinked networks, which fundamentally hinder the recyclability and reprocessability of SBR, resulting in resource waste and environmental burdens. In this work, SBR was used as the starting material. Through epoxidation and subsequent hydrogenation, followed by an epoxy ring-opening reaction, 3-aminophenylboronic acid (m-APBA) was introduced into the polymer chains, constructing a novel hydrogenated SBR with reversible dynamic cross-linking characteristics (HESBR-APBA). The resulting material exhibits superior mechanical properties compared to conventional hydrogenated SBR (HSBR) without any external additives. Notably, the HE7.4SBR-0.75APBA sample achieved a tensile strength of up to 14 MPa and retained over 95% of its original strength after multiple reprocessing cycles, demonstrating excellent mechanical stability and reprocessability. This study provides an effective molecular design strategy for balancing high mechanical performance and recyclability in hydrogenated SBR and offers new insights for developing reprocessable rubber material.

## 1. Introduction

Styrene–butadiene rubber (SBR), synthesized by a random copolymerization of butadiene and styrene, is one of the most widely used general-purpose synthetic rubbers [1]. It is characterized by excellent abrasion resistance, aging resistance, and satisfactory mechanical strength [2,3,4]. The unsaturated C=C bonds in SBR [5] endow the material with favorable elasticity [6] and mechanical properties [7]; however, these double bonds also make SBR susceptible to oxidative [8], ozone [9], and thermal degradation [10], leading to performance deterioration under demanding service conditions. Hydrogenation modification [11], which converts C=C bonds into C–C bonds, has been regarded as an effective approach to improving rubber performance and expanding its application scope [12].

Hydrogenated styrene–butadiene rubber (HSBR), obtained by hydrogenating SBR [13], exhibits enhanced thermal resistance [14], aging resistance [15], oil resistanc44e [16], chemical stability, and processing performance [17], as well as improved strain-induced crystallization behavior [18] and mechanical properties [1]. Liu et al. [19] developed an efficient catalytic system for hydrogenating the C=C bonds in SBR, achieving a hydrogenation degree of 95% without observable crosslinking in the absence of additives. Guo et al. [20] prepared a Pd/g-C_3_N_4_ catalyst using a simple chemical reduction method for the selective catalytic hydrogenation of SBR. This catalyst demonstrated exceptional activity under mild conditions, achieving a hydrogenation degree of 95%. Despite these advantages, the production of HSBR typically involves large amounts of chemical reagents, and in practical applications it is commonly cross-linked using sulfur or peroxides to form stable and permanent networks [21]. It is difficult to recycle or reprocess HSBR due to such irreversible cross-linked structures [22], leading to environmental pollution and resource waste [23].

In recent years, an increasing attention has been paid to reprocessable rubber materials [24]. By constructing dynamic crosslinked networks, waste rubber can retain favorable performances after simple reprocessing [25], thereby reducing environmental impact and improving resource utilization efficiency [26]. Dynamic covalent bonds [27,28] usually require complex chemical modification of polymer chains to introduce specific functional groups. Meanwhile, the enhancement of mechanical properties often relies on stronger covalent bonds or higher crosslink densities, which may compromise toughness and elasticity [3]. Consequently, developing high-performance recyclable rubber materials without sacrificing flexibility and elasticity remains a significant challenge [29]. Currently, dynamic cross-linked networks can be classified into covalent and noncovalent systems, including disulfide bonds [13], boronic ester bonds [30,31], imine bonds [14], and related interactions. Such structures have, to a certain extent, enabled self-healing and reprocessing properties in some materials. Liu et al. [32] designed ester-crosslinked rubber with high dynamic performances by transesterification, but the resulting rubber had a tensile strength of less than 3 MPa and an elongation at break below 400%. Cui et al. [33] designed a rubber with multiple reversible bonds (metal–ligand bonds, hydrogen bonds), exhibiting excellent self-healing efficiency, but its tensile strength remained below 10 MPa. Wajge et al. [34] enhanced the tensile strength of carboxylated nitrile rubber (XNBR) fourfold via dynamic coordination crosslinking with Fe^3+^, but the strength was still below 5 MPa. Cui et al. [4] designed a dual cross-linked network with imine bonds and metal–ligand bonds (copper–imine) to prepare self-healing, recyclable rubber, but its tensile strength was only 1 MPa. Thus, crosslinked networks in rubber based on dynamic bonds can impart certain recyclability and self-healing abilities, but enhancing mechanical performances remains a key issue [20].

In this study, SBR was used as the raw material. Through an epoxide ring-opening reaction followed by a hydrogenation process, 3-aminophenylboronic acid (m-APBA) was introduced into the polymer chains to construct a dynamic cross-linked network, yielding a reprocessable hydrogenated epoxidized styrene–butadiene rubber (HESBR-APBA). The effects of epoxidation and ring-opening conditions on reprocessability and mechanical properties were investigated. HESBR-APBA with a dual dynamic crosslinked network—composed of dynamic boronic ester bonds and reversible hydrogen bonds—was successfully constructed. Without the addition of any auxiliary agents, the resulting rubber material exhibits excellent initial mechanical properties and retains over 95% of its performance after reprocessing. This work provides new insights for the design and application of reprocessable rubber materials.

## 2. Materials and Methods

### 2.1. Materials

Styrene–butadiene rubber (SBR1502E) was purchased from Qilu Petrochemical Company, China Petroleum (Zibo, China). Xylene (analytical grade), anhydrous ethanol (analytical grade), and tetrahydrofuran (THF, C_4_H_8_O, analytical grade) were purchased from Fuyu Fine Chemical Co., Ltd. (Tianjin, China). Anhydrous formic acid (HCOOH, analytical grade) was purchased from Fuchen Chemical Reagent Co., Ltd. (Tianjin, China). Hydrogen peroxide (H_2_O_2_, 30 wt%) was purchased from Sinopharm Chemical Reagent Co., Ltd. (Beijing, China). Sodium hydroxide (NaOH, analytical grade) was purchased from Beijing Chemical Works (Beijing, China). 3-Aminophenylboronic acid (m-APBA, C_6_H_8_BNO_2_, 98%) was purchased from J&K Scientific Ltd. (Beijing, China). Nitrogen (N_2_, >99%) and hydrogen (H_2_, >99%) were purchased from Yong Sheng Gas Technology Co., Ltd. (Beijing, China). The hydrogenation catalyst was prepared in our laboratory.

### 2.2. Synthesis of Hydrogenated Epoxidized Styrene–Butadiene Rubber (HESBR)

Synthesis of Epoxidized Styrene–Butadiene Rubber (ESBR): First, SBR1502 was dissolved in xylene to prepare a rubber solution. A predetermined amount of this solution was then transferred to a three-necked flask. A mixture of anhydrous formic acid and hydrogen peroxide in a specific molar ratio was added dropwise to the rubber solution with stirring. The epoxidation reaction was conducted at 40 °C with stirring at 300 rpm for 6 h. Subsequently, the reaction mixture was neutralized by adding a predetermined amount of sodium hydroxide, followed by stirring for an additional 20 min. The product was precipitated, washed thoroughly with anhydrous ethanol, and dried in an oven at 60 °C for 24 h to obtain epoxidized SBR (designated as ESBR1502).

Synthesis of Hydrogenated Epoxidized Styrene–Butadiene Rubber (HESBR): The epoxidation ring-opening reaction conditions, material ratios, and post-treatment processes involved in this study were independently developed based on our preliminary explorations. Therefore, no references regarding the synthesis steps are cited in the manuscript. First, the self-prepared ESBR1502 was dissolved in xylene to prepare a rubber solution. A predetermined amount of this solution was then transferred to a high-pressure reactor, followed by the addition of a specified amount of the self-prepared hydrogenation catalyst. The reactor was purged three times with nitrogen and subsequently three times with hydrogen. The hydrogenation reaction was conducted at 90 °C under a hydrogen pressure of 3 MPa, with stirring at 200 rpm for 6 h. Upon completion, the product was precipitated, washed thoroughly with anhydrous ethanol, and dried in an oven at 60 °C for 24 h to obtain hydrogenated epoxidized SBR (designated as HESBR1502, generically as HExSBR, where x represents the epoxidation degree).

### 2.3. Synthesis of Hydrogenated Epoxidized Styrene–Butadiene Rubber Modified with 3-Aminophenylboronic Acid (HESBR-APBA)

A predetermined amount of 3-aminophenylboronic acid (m-APBA) dissolved in tetrahydrofuran (THF) was added to a three-necked flask. Under a nitrogen atmosphere, at 60 °C and with stirring at 300 rpm, a THF solution of HESBR1502 was added dropwise to the flask. After the complete addition, the ring-opening reaction was allowed to proceed for 6 h. The final product was precipitated, washed thoroughly with anhydrous ethanol, and dried in an oven at 60 °C for 24 h, yielding HESBR-APBA. Based on the epoxidation degree of the starting rubber and the molar ratio of the added m-APBA to epoxy groups, the samples are named as HExSBR-yAPBA, where x is the epoxidation degree and y is the aforementioned molar ratio.

### 2.4. Characterization

#### 2.4.1. Fourier Transform Infrared Spectroscopy (FTIR)

Samples were scanned using ATR-FTIR on a Bruker Alpha II (Bruker, Karlsruhe, Germany) to characterize the functional group structures of HESBR with different epoxidation degrees. Specifically, in ATR mode, HESBR-APBA samples (30 °C to 210 °C) were recorded in the range of 4000 cm^−1^ to 500 cm^−1^ to characterize the formation of dynamic bonds.

#### 2.4.2. ^1^H Nuclear Magnetic Resonance (^1^H NMR)

Samples were dissolved in deuterated chloroform and analyzed using ^1^H NMR at 400 MHz on an AVANCE III HD 400 (Bruker, Germany). The hydrogenation degree and $X=\frac{A_{epoxy}}{A_{epoxy}+A_{\left(1,2\right)}+A_{\left(1,4\right)}}\times 100$ epoxidation degree of HESBR were calculated based on the integral areas of the peaks. The epoxidation degree was calculated using Equation (1).

Specifically, in the ^1^H NMR spectrum of HESBR, *A_epoxy_* is the integral area due to epoxy groups, *A*_(1,2)_ is due to 1,2-vinyl butadiene units, and *A*_(1,4)_ is due to 1,4-cis/trans butadiene units. The epoxidation degree was calculated using the equation based on the integral ratios in the ^1^H NMR spectrum.

(1) $$X=\frac{A_{epoxy}}{A_{epoxy}+A_{\left(1,2\right)}+A_{\left(1,4\right)}}\times 100$$

#### 2.4.3. Synchrotron Radiation Wide-Angle X-Ray Diffraction (WAXD)

First, the pressed raw rubber was cut into dumbbell-shaped specimens and fixed onto a custom-built stretching device. The device stretched the specimens at a preset constant speed and stopped immediately upon reaching a specific strain value. Using the U7B beamline of the synchrotron radiation facility at the Institute of High Energy Physics, Chinese Academy of Sciences, as the light source, with a wavelength set to 1.54 Å and an exposure time of 20 s, 2D Wide-Angle X-ray Diffraction (WAXD) images were rapidly captured. The images were analyzed using FIT2D software; after background subtraction, the data was integrated to obtain q-I curves, which were then converted to 2θ-I curves using Equation (2) via q-θ transformation. This procedure was used to characterize the tensile crystallization of HSBR with different styrene contents under various strains.

(2) $$\sin\theta =\frac{q\lambda }{4\pi }$$

#### 2.4.4. Cross-Linking Density (V_e_) Test

The test was conducted using the swelling method. A specimen with a mass of *m*1 was placed in a toluene solution. After 72 h of immersion, the specimen was taken out, and the toluene on its surface was wiped off. It was then weighed again to obtain *m*2. The value of *V*e was calculated using the Flory-Huggins formula, i.e., Equation (3).

(3) $$Ve=\frac{1}{2Mc}$$

Here, *M*c is the molecular weight between crosslinks, which can be calculated using Equation (4)

(4) $$Mc=\frac{-\rho_{p}V_{s}\left[{\phi_{p}}^{\frac{1}{3}}-{\phi_{p}}^{\frac{1}{2}}\right]}{\ln(1-\phi_{p})+\phi_{p}+x{\phi_{p}}^{2}}$$

Here, *Vs* is the molar volume of toluene, and *ϕ*_p_ is the volume fraction of the polymer in the swollen state, which can be calculated using Equation (5).

(5) $$\phi_{p}={\left[1+\frac{\rho_{p}m_{2}}{\rho_{s}m_{1}}-\frac{\rho_{p}}{\rho_{s}}\right]}^{-1}$$

Here, *ρ_s_* is the density of toluene, and *ρ_p_* is the density of the sample. The interaction parameter χ can be calculated using Equation (6):

(6) $$x=\beta +\frac{V_{s}}{RT{\left(\delta_{S}-\delta_{P}\right)}^{2}}$$

#### 2.4.5. Differential Scanning Calorimetry (DSC)

Performed on a TGA/DSC1 (Mettler Toledo, Zurich, Switzerland) under N_2_ atmosphere, with a heating rate of 10 °C/min from −60 °C to 60 °C, to characterize the glass transition temperature (*T_g_*) of HESBR and HESBR-APBA.

#### 2.4.6. Thermogravimetric Analysis (TGA)

Performed on a TGA1 (Mettler Toledo, Switzerland) using an automatic sampler, under N_2_ atmosphere with a heating rate of 10 °C/min, measuring mass loss from 30 °C to 800 °C.

#### 2.4.7. Tensile Properties Test

The stress–strain curve test was conducted in accordance with the GB528-2009 standard [35]. First, the 1 mm thick sheet was cut into small dumbbell-shaped specimens measuring 75 mm × 13 mm, with a gauge length of 35 mm. Five specimens were taken from each sample group. Conducted on an AI-7000S1 (Gotech, Taichung, Taiwan, China) universal tensile testing machine following the GB528-2009 standard, to characterize the tensile properties of HESBR and HESBR-APBA.

#### 2.4.8. Dynamic Mechanical Analysis (DMA)

Performed on a TA Q800 (Mettler Toledo, Greifensee, Switzerland). The test conditions were as follows: frequency 10 Hz, strain 0.2%, temperature range −100 °C to 80 °C, heating rate 3 °C/min.

#### 2.4.9. Reprocessing Performance Test

After being physically crushed and passed through a roller, HESBR-APBA was hot-pressed into sheets at 155 °C. Tensile testing was conducted on an AI-7000S1 (Gotech, Taiwan, China) universal tensile testing machine following the GB528-2009 standard to characterize the reprocessing performance of HESBR-APBA.

#### 2.4.10. Oil Resistance Test

Oil resistance testing was conducted in accordance with the GB/T1690-2010 standard [36]. In the experiment, IRM901 and IRM903 were selected as the test oils. The rubber sheets were first immersed in an oil bath and then placed in a constant-temperature oven at 150 °C for 24 h of aging. The calculation formula is given in Equations (2)–(4), where W_0_ represents the mass of the film before swelling, and W_s_ represents the mass after swelling.

(7) $$\mathrm{Swelling}\;\mathrm{ratios}\%=\frac{W_{s}-W_{0}}{W_{0}}\times 100\%$$

## 3. Results and Discussion

### 3.1. Structure and Properties of HESBR

**Figure 1:**
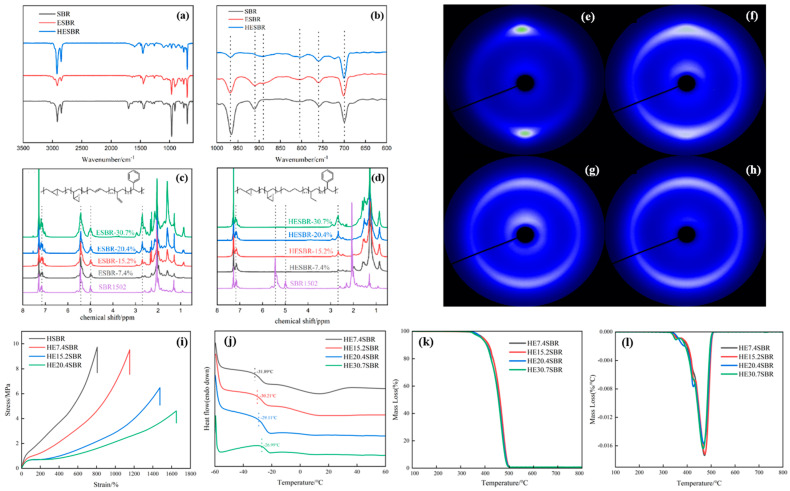
Characterization of SBR, ESBR, and HESBR with varying epoxidation degrees. (**a**) ATR-FTIR spectra in the range of 600–3500 cm^−1^. (**b**) ATR-FTIR spectra in the range of 600–1000 cm^−1^. (**c**) ^1^H NMR spectrum of ESBR. (**d**) ^1^H NMR spectrum of HESBR. (**e**–**h**) Instantaneous 2D WAXD patterns at a tensile ratio of 700% for (**e**) HSBR, (**f**) HE7.4SBR, (**g**) HE15.2SBR, and (**h**) HE20.4SBR. (**i**) Stress–strain curves of HSBR and HExSBR (**j**) DSC curves. (**k**) TGA curves. (**l**) DTG curves. All thermal analysis and 2D WAXD data are for HESBR samples with four different epoxidation degrees.

The ATR-FTIR spectra of SBR, ESBR and HESBR are shown in Figure 1a,b. The characteristic peaks in the spectra of HESBR are assigned as follows: the peaks at 700 and 760 cm^−1^ correspond to the benzene ring vibrations of the polystyrene segments, which is consistent with the characteristic peak positions of styrene-based rubbers reported in the literature [37]; the peak at 887 cm^−1^ represents the epoxy group; the peaks at 912, 965, and 803 cm^−1^ are attributed to the vinyl-1,2-, trans-1,4-, and cis-1,4-C=C vibrations of the polybutadiene units, respectively. Compared to SBR, both ESBR and HESBR exhibit a new characteristic peak at 887 cm^−1^, confirming the successful introduction of epoxy groups. Furthermore, the characteristic peaks of the epoxy group and styrene remain unchanged after hydrogenation, indicating that the hydrogenation process selectively saturated the C=C bonds without affecting the epoxy or styrene structures.

Figure 1c presents the ^1^H NMR spectrum of ESBR, while (d) shows that of HESBR. As shown in Figure 1c, with increasing amounts of formic acid and hydrogen peroxide during epoxidation, the characteristic peaks of styrene remain constant, the signals from -C=C- decrease, the styrene proton peaks (chemical shift 6.5–7.2 ppm) remained unchanged, while the characteristic peaks of -C=C- double bonds (chemical shift 5.0–5.5 ppm) gradually decreased, and the characteristic peaks of epoxy groups (chemical shift 2.7–3.0 ppm) [38] gradually intensified. Figure 1d reveals that upon hydrogenation of ESBR to form HESBR, the styrene signal remains, whereas the signals for both 1,2- and 1,4-C=C- disappear completely. Concurrently, the signals in the region of δ = 0.5–2.5 ppm, corresponding to saturated hydrocarbon protons, strengthen, confirming the conversion of double bonds to single bonds. The persistence of the epoxy group signal confirms that the epoxidation degree remains consistent before and after hydrogenation.

Figure 1e–h display the instantaneous 2D WAXD patterns at a tensile strain of 700%. HSBR (Figure 1e) shows the most distinct crystalline diffraction spots, indicating a pronounced strain-induced crystallization behavior and a higher crystallinity at this strain. According to literature reports, the orientation of molecular chains along the stress direction and the formation of ordered structures during the stretching of rubber are the reasons for the appearance of sharp diffraction spots [39]. As the epoxidation degree increases, the diffraction spots progressively degenerate into arcs. At an epoxidation degree of 7.4% (Figure 1f), relatively distinct spots are still observable. However, at 15.2% (Figure 1g), the spots completely transform into arcs, though these arcs remain sharper than those of the sample with 20.4% epoxidation (Figure 1h), suggesting that the crystallinity of HE15.2SBR is higher than that of HE20.4SBR.

Figure 1i presents the stress–strain curves of HSBR and HESBR with epoxidation degrees of 7.4%, 15.2%, and 20.4%. It can be observed that as the epoxy content increases, the tensile strength of the materials exhibits a decreasing trend, while the elongation at break shows a gradual increase. Among them, HSBR demonstrates the highest tensile strength and the lowest elongation at break, whereas HESBR-E20.4 displays the lowest tensile strength and the highest elongation at break. This phenomenon can be attributed to the disruption of the -(CH_2_)n- chain segments in the molecular structure by epoxy groups with increasing epoxidation degree, leading to reduced crystalline phase formation during stretching and consequently diminished interchain cohesion. As a result, the tensile strength decreases. However, due to the reduced interchain cohesion, the polymer chains gain enhanced extensibility under external force, thereby increasing the elongation at break. These findings are consistent with the instantaneous 2D WAXD image acquisition results obtained for the aforementioned rubbers.

Figure 1j–l present the thermal analysis results. The DSC curves in Figure 1j show that the Tg of HESBR increases with the epoxidation degree. This rise is attributed to enhanced intermolecular interactions introduced by the epoxy groups and the suppression of crystallization. The relatively regular polyethylene-like segments in HSBR can crystallize, but epoxidation introduces structural irregularity, which first hinders and, at higher levels, completely eliminates crystallization. The TGA and DTG curves in Figure 1k and Figure 1l, respectively, demonstrate that a higher epoxidation degree leads to a lower initial decomposition temperature and a lower maximum decomposition rate temperature. The progressive increase in the intensity of the DTG shoulder near 425 °C correlates with the thermal decomposition of the epoxy groups. Overall, increased epoxidation degrades the thermal stability of HESBR, as evidenced by reduced decomposition temperatures.

### 3.2. Structural Characterization of HESBR-APBA

Ring-opening of HESBR with four different epoxidation degrees was carried out while regulating the ratio of 3-aminophenylboronic acid to epoxy groups, leading to the results presented in Table 1. First, ring-opening experiments were performed on four HESBR samples with different epoxidation degrees under identical reaction conditions. Among them, Sample 4, with the highest epoxidation degree, led to severe over-crosslinking during the reaction with m-APBA, resulting in substantial gel formation. Therefore, the mechanical properties, crosslinking density, and reprocessing performance of the first three samples were evaluated. The results indicate that HE7.4SBR exhibits the best overall performance after the ring-opening reaction. Consequently, this sample was selected for further study, in which the molar ratio of epoxy groups to 3-aminophenylboronic acid was systematically varied to identify the optimal reaction conditions.

**Figure 2:**
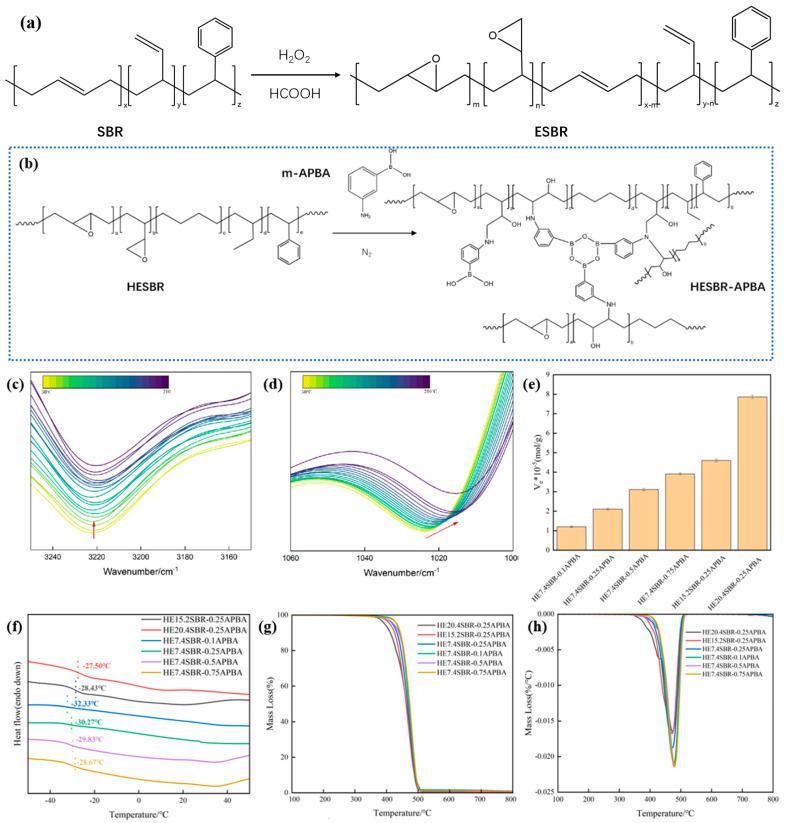
(**a**) Synthesis process of ESBR; (**b**)HESBR-APBA preparation flow chart and Schematic diagram of borate ester bond in the cross-linked network structure; (**c**,**d**) Variable-temperature infrared spectrum; (**e**) Crosslinking density histogram; (**f**) DSC curves; (**g**) TGA and (**h**) DTG curves of HESBR-APBA.

Figure 2a,b schematically illustrates the reaction mechanism for preparing ESBR from SBR, and HESBR-APBA from HESBR and m-APBA, and the resulting cross-linked network containing dynamic boronic ester bonds. Due to its cross-linked nature, the structure of HESBR-APBA is not amenable to direct NMR characterization. Therefore, variable-temperature infrared spectroscopy was employed to probe the dynamic features of the network. Figure 2c, d present the variable-temperature FTIR spectra of HESBR-APBA. In Figure 2e, upon heating from 30 °C to 210 °C (The arrow indicates the direction of temperature increase), the characteristic peak at 1023 cm^−1^, assigned to the B–O stretching vibration in boronic ester bonds, gradually shifts to lower wavenumbers and decreases in intensity. This indicates the thermally induced cleavage of the dynamic B–O bonds, confirming their successful incorporation into the polymer network. Concurrently, Figure 2d shows the attenuation of the broad absorption band around 3220 cm^−1^, which is associated with the stretching vibrations of hydroxyl (-OH) and amino (-NH) groups involved in hydrogen bonding. This weakening suggests the dissociation of hydrogen bonds at elevated temperatures. Figure 2e shows the crosslinking density of various HESBR-APBA samples. The crosslinking density was calculated according to Equations (3)–(6). For the HE7.4SBR-xAPBA series, the crosslinking density increases with a higher m-APBA feed ratio. Similarly, for the HExSBR-0.25APBA series, it increases with the epoxidation degree. The highest crosslinking density is observed for HE20.4SBR-0.25APBA, which exhibits over-crosslinking and consequently the poorest mechanical properties. This trend is rationalized by the crosslinking mechanism: a higher m-APBA concentration or a greater number of epoxy groups leads to a higher ring-opening reaction rate and extent, thereby increasing the crosslinking density. However, excessive crosslinking adversely affects mechanical and processing properties.

Figure 2f displays the DSC heating curves. The *T_g_* of HESBR-0.25APBA is slightly higher than that of the precursor HESBR. This modest increase results from a balance between two opposing factors: the enhanced chain rigidity imparted by the cross-linked network tends to raise *T_g_*, while the consumption of rigidifying epoxy groups during network formation tends to lower it. Within the HE7.4SBR-xAPBA series, *T_g_* generally increases with the increase in m-APBA content, as the effect of the cross-linked network becomes dominant. The exception is HE7.4SBR-0.1APBA, whose *T_g_* is lower than that of HE7.4SBR, suggesting that its sparse network is insufficient to compensate for the *T_g_* reduction caused by epoxy group loss. Figure 2g,h present the TGA and DTG curves, respectively. Since the dynamic hydrogen bonds and boronic ester bonds dissociate well below 300 °C [40], the thermal stability of HESBR-APBA above this temperature is primarily governed by the remaining epoxy groups and the polymer backbone. For the HExSBR-0.25APBA series, a higher epoxidation degree leads to a lower initial decomposition temperature, while the maximum decomposition temperature remains similar. The persistent DTG shoulder near 425 °C for HE20.4SBR-0.25APBA indicates a significant residual epoxy content. In contrast, for the HE7.4SBR-xAPBA series, a higher m-APBA content results in a higher initial decomposition temperature and a greater maximum decomposition rate, indicating improved thermal stability. The T5% of HE7.4SBR-0.25APBA is 458 °C, which is higher than that of HE20.4SBR-0.25APBA (450 °C), indicating that a lower degree of epoxidation enhances thermal stability. Furthermore, the char residues are 12.5% and 10.8%, respectively. These data collectively indicate that HE7.4SBR-0.25APBA possesses slightly better thermal stability. Overall, the ring-opening modification enhances the thermal resistance of HESBR, with the optimal HE7.4SBR-APBA sample exhibiting stability comparable to that of HSBR.

### 3.3. Mechanical Properties of HESBR and HESBR-APBA

**Figure 3:**
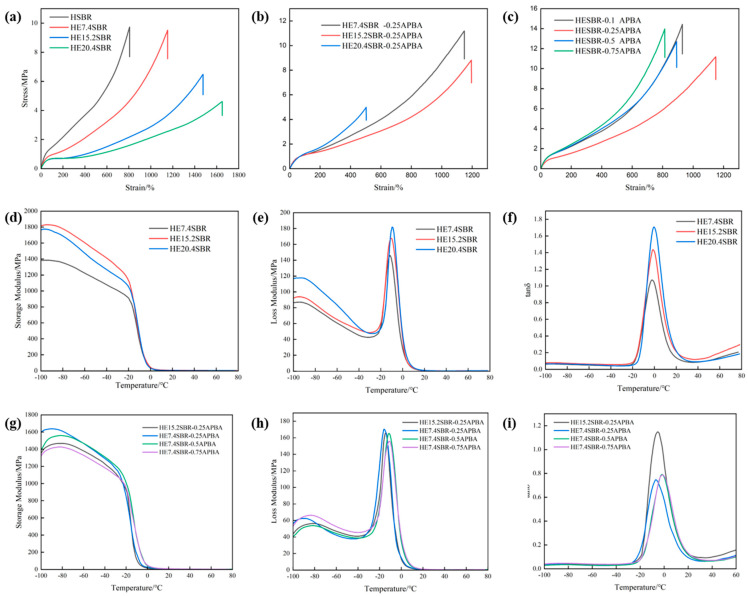
Stress–strain curves of (**a**) HSBR and HExSBR; (**b**) HExSBR-APBA; (**c**) HE7.4SBR-APBA; (**d**) Storage Modulus; (**e**) Loss Modulus; (**f**) tanδ curves of HExSBR; (**g**) Storage modulus; (**h**) Loss Modulus; (**i**) tanδ curves of HESBR-APBA.

Figure 3a presents the stress–strain curves of HSBR and HESBR with epoxidation degrees of 7.4%, 15.2%, and 20.4%. With increasing epoxy content, the tensile strength decreases while the elongation at break increases. This is attributed to the disruption of the regular polyethylene-like –(CH_2_)_n_– segments by the introduced epoxy groups, which suppresses strain-induced crystallization. Fewer crystalline phases form during stretching, reducing interchain cohesion and tensile strength. Conversely, the reduced cohesion enhances chain extensibility, leading to higher elongation at break. These findings are consistent with the crystallization analysis, confirming that epoxy groups disrupt the inherent crystallization ability of HSBR and consequently diminish its mechanical performance. Figure 3b shows the stress–strain curves of HExSBR-0.25APBA with different epoxidation degrees after ring-opening. HE30.7SBR-0.25APBA is omitted due to severe gelation caused by over-crosslinking. The mechanical properties of HESBR-APBA are governed by a combination of the residual strain-induced crystallization of HSBR and the newly formed dynamic cross-linked network. As shown, HE7.4SBR-0.25APBA exhibits the best mechanical performance. The decline of mechanical properties for samples with higher epoxidation degrees results from two factors: first, the progressive weakening of strain-induced crystallization, and second, the increased crosslink density after ring-opening. Although the network provides reinforcement, excessive crosslinking ultimately degrades mechanical properties. Compared to HESBR, HESBR-0.25APBA shows significantly improved tensile strength but reduced elongation at break. The cross-linked network enhances strength but restricts chain mobility, thereby decreasing extensibility.

Figure 3d displays the storage modulus (E′) curves of HESBR. The modulus increases with epoxidation degree because the rigid epoxy groups disrupt the flexible –(CH_2_)_n_– segments, increasing the overall rigidity of the rubber. Figure 3e shows the loss modulus (E″) curves. As the epoxidation degree rises, the curves shift to higher temperatures and the peak values increase, indicating a rise in the glass transition temperature (*T_g_*), consistent with DSC results. More epoxy groups enhance chain rigidity and internal friction, leading to higher E″ peak values.

To investigate the effects of m-APBA content and epoxidation degree on dynamic mechanical properties, HE7.4SBR-0.25APBA, HE7.4SBR-0.5APBA, and HE15.2SBR-0.25APBA were compared. Figure 3g presents the storage modulus curves. With increasing m-APBA content, E′ decreases, primarily due to the competing effects of crosslink density and epoxy group consumption. Higher m-APBA content increases crosslink density, restricting chain mobility at elevated temperatures, as evidenced by a rightward curve shift and increased *T_g_*. This effect is more pronounced at higher epoxidation degrees because more epoxy groups are available as crosslinking sites, leading to greater crosslink density for a given m-APBA amount. Additionally, increased m-APBA content reduces the curve height, indicating enhanced material rigidity and reduced flexibility, making the material more brittle. From the loss modulus curves in Figure 3h, it can be seen that with higher m-APBA content and epoxidation degree, the loss peak shifts to higher temperatures, reflecting improved thermal stability. Increased crosslink density restricts chain movement, thereby raising *T_g_*. A higher epoxidation degree provides more reactive sites, resulting in greater crosslink density and a more significant increase for *T_g_*, as seen in the comparison between HE15.2SBR-0.25APBA and HE7.4SBR-0.25APBA. The cross-linked structure hinders the disentanglement and energy dissipation of long chain segments near *T_g_*, reducing the number of participating segments and slightly lowering the E″ peak intensity.

The loss factor (tan δ) curves in Figure 3i show that with increasing m-APBA content and epoxidation degree, the tan δ peak temperature rises continuously, and the peak becomes narrower and sharper, indicating changes in the internal structure and viscoelastic behavior. Higher crosslink density imposes greater constraints on chain mobility, leading to more coordinated segmental motion. The number of chains that can disentangle and dissipate energy near *T_g_* decreases, resulting in a narrower peak. A higher epoxidation degree promotes a more complete reaction with m-APBA, forming a more uniform network and a sharper tan δ peak.

**Figure 4:**
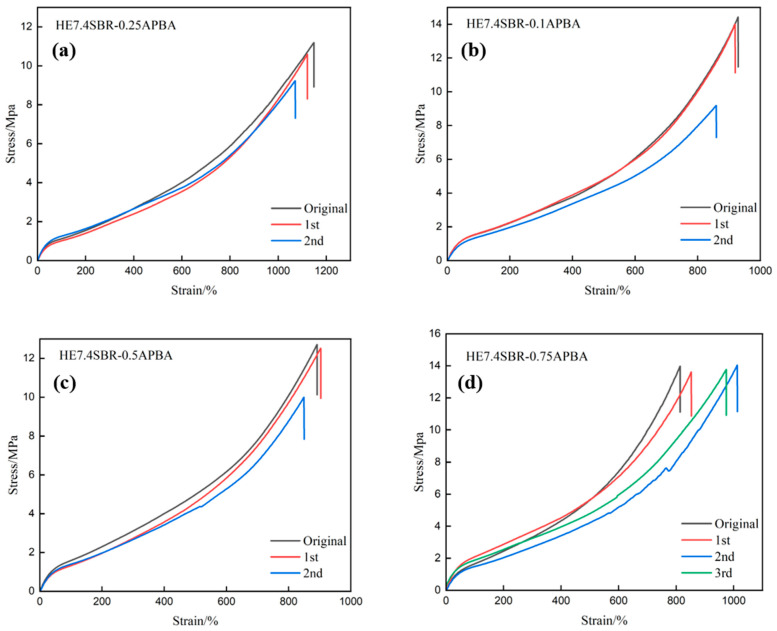
Stress–strain curves of (**a**) HE7.4SBR-0.25APBA; (**b**) HE7.4SBR-0.1APBA; (**c**) HE7.4SBR-0.5APBA; (**d**) HE7.4SBR-0.75APBA after heat pressing treatment.

Figure 4a shows the stress–strain curves of HE7.4SBR-0.25APBA after repeated reprocessing cycles. The material exhibits good reprocessability, maintaining a tensile strength of 10.29 MPa after three cycles, with a retention rate above 90%. The stress–strain curves of HE7.4SBR-0.1APBA and HE7.4SBR-0.5APBA are presented in Figure 4b and Figure 4c, respectively. HE7.4SBR-0.1APBA shows excellent initial mechanical properties, attributable to the reinforcing effect of its initially sparse cross-linked network. After the first reprocessing cycle, it retains more than 95% of both tensile strength and elongation at break. However, a significant decrease in mechanical properties is observed after the second cycle, which can be ascribed to the limited m-APBA content resulting in an underdeveloped dynamic network that degrades upon repeated processing. HE7.4SBR-0.5APBA shows a similar reprocessing trend—good performance after one cycle but a decline after two cycles. Its initial properties are inferior to those of HE7.4SBR-0.1APBA because the denser cross-linked network disrupts the regularity of the polymer chains. Figure 4d displays the stress–strain curves of HE7.4SBR-0.75APBA, which demonstrates the most outstanding reprocessability. Its tensile strength retention remains above 95% even after three reprocessing cycles. The slight increase in elongation at break after reprocessing may be attributed to chain scission acting as an internal plasticizer. The excellent reprocessability of this material derives from the dual dynamic cross-linked network structure comprising dynamic boronic ester bonds and reversible hydrogen bonds, as described above. It has been demonstrated in the literature [41,42,43] that cross-linked systems featuring such a structure exhibit dynamic network behavior.

In summary, HE7.4SBR-0.75APBA exhibits the best combination of mechanical properties and reprocessability, achieving a tensile strength of 14 MPa and retaining this performance level after three reprocessing cycles. Overall, HESBR-APBA maintains a mechanical strength retention rate exceeding 90% after a single reprocessing cycle, significantly higher than that of HESBR, successfully meeting the design objective of reprocessability.

### 3.4. Oil Resistance of HESBR and HESBR-APBA

**Figure 5:**
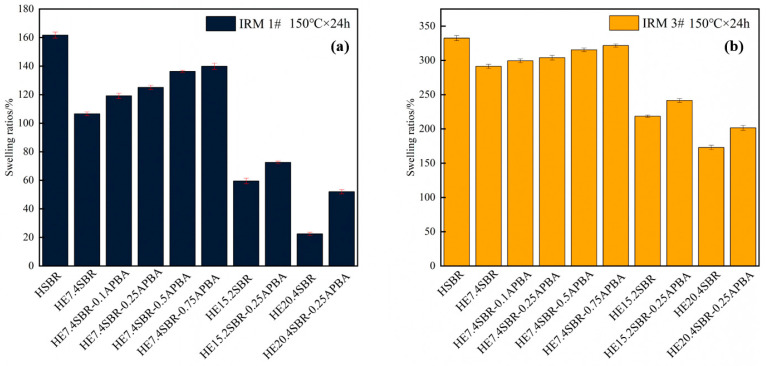
Oil resistance of different rubber (**a**) IRM1 oil (**b**) IRM3 oil.

The oil resistance of different rubber samples in IRM1 and IRM3 oils is shown in Figure 5, which exhibits similar trends. Preliminary experiments showed that after 72 h of immersion, the mass of the samples showed no significant change, indicating that swelling equilibrium had been reached. Compared to HSBR, the oil resistance of HESBR improves progressively with increasing epoxidation degree, owing to the enhanced polarity introduced by the epoxy groups. In contrast, the oil resistance of HESBR-APBA is inferior to that of its HESBR precursor and gradually decreases with higher m-APBA content, though it remains superior to HSBR. This is because the oil resistance of HESBR-APBA depends on a balance between the polarity of the remaining epoxy groups and the restraint provided by the cross-linked network. Since network formation consumes epoxy groups, the contribution from the cross-linked structure to oil swelling resistance is less effective than that of the original polar epoxy groups. Consequently, while HESBR-APBA shows weaker oil resistance than HESBR, it still performs significantly better than HSBR.

## 4. Conclusions

Through an oxidative ring-opening reaction, m-APBA was introduced into the HESBR molecular chain, successfully constructing HESBR-APBA with a dual dynamic cross-linked network comprising both dynamic boronic ester bonds and reversible hydrogen bonds.

The research results indicate that as the epoxidation degree of HESBR increases, the cross-linking density of the system significantly rises. Excessive cross-linking restricts the movement of rubber chain segments, leading to a gradual decline in the material’s mechanical properties and reprocessability, accompanied by an increase in *T_g_*. In contrast, with an increase in the ring-opening ratio of epoxy groups, the cross-linking density continues to grow. The mechanical properties of the material show a trend of initial decline followed by improvement, while reprocessability is significantly enhanced, *T_g_* increases and oil resistance decreases.

Notably, the HE7.4SBR-0.75APBA sample exhibits relatively excellent comprehensive performance, benefiting from strain-induced crystallization, the tensile strength reaches 14 MPa and maintaining a tensile strength retention rate above 95% even after multiple reprocessing cycles. This achieves an effective balance among mechanical properties, reprocessability, and thermal performance within the HESBR-APBA system, providing a feasible strategy for the molecular structure design of high-performance, recyclable styrene–butadiene rubber materials.

## Figures and Tables

**Table 1:** Preparation process conditions of HESBR-APBA.

No.	Epoxidation Degree/%	HD/%	Epoxy:m-APBA (mol:mol)
1	7.4	99	1:0.25
2	15.2	99	1:0.25
3	20.4	99	1:0.25
4	30.7	99	1:0.25
5	7.4	99	1:0.10
6	7.4	99	1:0.50
7	7.4	99	1:0.75

## Data Availability

The original contributions presented in this study are included in the article. Further inquiries can be directed to the corresponding author.
